# Dataset of consumer-based activity trackers as a tool for physical activity monitoring in epidemiological studies during the COVID-19 Pandemic

**DOI:** 10.1016/j.dib.2022.108003

**Published:** 2022-03-01

**Authors:** André Henriksen, Erlend Johannessen, Gunnar Hartvigsen, Sameline Grimsgaard, Laila Arnesdatter Hopstock

**Affiliations:** a Department of Computer Science, UiT The Arctic University of Norway, Tromsø, Norway; b Department of Community Medicine, UiT The Arctic University of Norway, Tromsø, Norway

**Keywords:** Energy expenditure, Steps, Smart watch, Fitness tracker, Public health, Lockdown, SARS-CoV-2, Wearables

## Abstract

Physical activity (PA) data were downloaded from 113 participants who owned a Garmin or Fitbit activity tracker in 2019 and 2020. Upon participant authorization, data were automatically downloaded from the Garmin and Fitbit cloud storages. The mSpider tool, a solution for automatic and continuous data extraction from activity tracker providers, were used to download participant data. Available data are daily averages by year, as well as monthly averages between 2019 and 2020, for steps, activity energy expenditure (AEE), total energy expenditure (TEE), moderate-to-vigorous physical activity (MVPA), light PA (LPA), moderate PA (MPA), vigorous PA (VPA), and sedentary time. In addition, March 2020 was divided in two, giving the daily average before and after the Norwegian COVID-19 lockdown date. Raw daily values for these variables are also included in a separate file. In addition, daily values for non-wear time are also include as raw data.

In a previous study, differences between months, i.e., comparing 2019 with 2020 for months between March to December, were analysed for steps, MVPA, and AEE [1]. Further insights may be achieved by exploring other variables. This includes: (1) monthly averages for TEE, LPA, MPA, VPA, and sedentary time, (2) yearly averages (2019 and 2020) for steps, MVPA, TEE, AEE, LPA, MPA, VPA, and sedentary time (3) monthly average for steps, MVPA, TEE, AEE, LPA, MPA, VPA, and sedentary time for January, February, and March 2019, as well as March 2020. Additional analysis can also be conducted on the raw data.

## Specifications Table

**Table:** 

Subject	Health informatics
Specific subject area	Change in physical activity levels during Norwegian COVID-19 lockdown.
Type of data	Table
How the data were acquired	Data were downloaded directly from the Fitbit and Garmin cloud storages. The mSpider tool, a solution for automatic and continuous data extraction from activity tracker providers, were used to extract historic data as well as future data from the day of participant registration. Upon registration, participants were asked to authorize access to the mSpider tool, by using open authentication provided by the vendor system (i.e., Fitbit/Garmin).
Data format	Raw, Analysed
Description of data collection	Participants were recruited through Norwegian media outlets. People who already owned an activity tracker from Garmin or Fitbit were eligible for inclusion. Two years of data (2019-2020) were automatically downloaded from consenting participants by directly accessing the Garmin and Fitbit cloud storages. The mSpider tool described in Henriksen et al. (2021) [1] were used to automate data downloading. Data were stored anonymized after download.
Data source location	Institution: UiT The Arctic University of Norway City/Town/Region: Tromsø/-/Troms Country: Norway Latitude and longitude for collected samples/data: Norway
Data accessibility	Repository name: DataverseNO Data identification number: doi: 10.18710/TGGCSZ, Direct URL to data: https://dataverse.no/dataset.xhtml?persistentId=doi:10.18710/TGGCSZ [2]
Related research article	A. Henriksen. E. Johannessen, G. Hartvigsen, S. Grimsgaard, L. A. Hopstock, Consumer-Based Activity Trackers as a Tool for Physical Activity Monitoring in Epidemiological Studies During the COVID-19 Pandemic: Development and Usability Study. JMIR Public Health Surveill, 2021. 7(4): p. e23806. doi: https://doi.org/10.2196/23806[1]

## Value of the Data

• These data are useful because they give insight in how physical activity levels changed in the population due to the Norwegian COVID-19 lockdown.
• Researchers who wish to confirm results or perform more complex analysis on variables used in the affiliated publication, may benefit from these data.
• The dataset also includes monthly averages for TEE, LPA, MPA, VPA, and sedentary time. These were not included or addressed in the affiliate publication.
• Further insights may be achieved by doing more complex statistical analysis on the monthly averages presented in the affiliated publication (for Steps, MVPA, AEE).
• Further insights may be achieved by analysing variables not used in the affiliated publication. These includes (for each participant): 1) activity tracker provider, 2) monthly averages for TEE, LPA, MPA, VPA, and sedentary time, 3) yearly average for 2019 and 2020 for steps, MVPA, TEE, AEE, LPA, MPA, VPA, and sedentary time, 4) monthly averages for steps, MVPA, TEE, AEE, LPA, MPA, VPA, and sedentary time for January, February, and March 2019, as well as March 2020.
• Further insight may be achieved by analysing the raw data which the analysed variables are based on. This includes daily values for steps, MVPA, TEE, AEE, LPA, MPA, and VPA, as well as sedentary time and non-wear time.

## 1 Data Description

Data were collected from 113 participants who shared their physical activity data using privately owned activity trackers (or smart watches) from Garmin and Fitbit Table 1. provides summary statistics of the 106 participants who responded to an anonymous questionnaire asking about height, weight, age, and gender. Individual participant characteristics were unable to be paired to their respective PA data.

**Table 1:** Participant characteristics.

Variable (n=106)	Mean (SD)	Ranges
Height in cm	173.5 (8.0)	158–194
Weight in kg	76.0 (14.3)	53.5–147.0
Body mass index (kg/m^2^)	25.2 (4.0)	18.3–50.3
Age in years (n=104)	40.6 (10.6)	21–69

**Gender (n=105)**	**Percentage**	

Males	43.8 (46)	Not applicable
Females	56.2 (59)	Not applicable

The dataset is stored at DataverseNO [2] and contains two files (“data.csv” and “data raw.csv”), in addition to a readme-file (“00_ReadMe.txt”) which describes the content of the files in the dataset.

The “data.csv” file contains 224 physical activity related variables, in addition to participant ID and provider name. The data consists of up to two years (2019-2020) of daily average values, grouped by month, for steps, total energy expenditure (TEE), activity energy expenditure (AEE), moderate-to-vigorous physical activity (MVPA), light PA (LPA), moderate PA (MPA), vigorous PA (VPA), and sedentary time. In addition to daily averages for each month, daily averages for 2019 and 2020, for each physical activity outcome, are also included. Finally, separate variables for the first and second half of March 2020 (before- and after the COVID-19 lockdown date in Norway in 2020) are included Table 2. gives a description of all variables included in the “data.csv” dataset.

**Table 2:** Variable description, data.csv.

#	Variable	Description
1	ID	Anonymized participant identified (1-113).
2	Provider	Provider name (Garmin/Fitbit).
3	2019-01-Steps	Average daily steps in January 2019.
4	2019-02-Steps	Average daily steps in February 2019.
5	2019-03-Steps	Average daily steps in March 2019.
6	2019-04-Steps	Average daily steps in April 2019.
7	2019-05-Steps	Average daily steps in May 2019.
8	2019-06-Steps	Average daily steps in June 2019.
9	2019-07-Steps	Average daily steps in July 2019.
10	2019-08-Steps	Average daily steps in August 2019.
11	2019-09-Steps	Average daily steps in September 2019.
12	2019-10-Steps	Average daily steps in October 2019.
13	2019-11-Steps	Average daily steps in November 2019.
14	2019-12-Steps	Average daily steps in December 2019.
15	2020-01-Steps	Average daily steps in January 2020.
16	2020-02-Steps	Average daily steps in February 2020.
17	2020-03-Steps	Average daily steps in March 2020.
18	2020-04-Steps	Average daily steps in April 2020.
19	2020-05-Steps	Average daily steps in May 2020.
20	2020-06-Steps	Average daily steps in June 2020.
21	2020-07-Steps	Average daily steps in July 2020.
22	2020-08-Steps	Average daily steps in August 2020.
23	2020-09-Steps	Average daily steps in September 2020.
24	2020-10-Steps	Average daily steps in October 2020.
25	2020-11-Steps	Average daily steps in November 2020.
26	2020-12-Steps	Average daily steps in December 2020.
27	2020-03-01-Steps	Average daily steps between March 1^st^-12^th^, 2020.
28	2020-03-13-Steps	Average daily steps between March 13^th^-31^st^, 2020.
29	2019-Steps	Average daily steps in 2019.
30	2020-Steps	Average daily steps in 2020.
31	2019-01-Mvpa	Average daily minutes of MVPA in January 2019.
32	2019-02-Mvpa	Average daily minutes of MVPA in February 2019.
33	2019-03-Mvpa	Average daily minutes of MVPA in March 2019.
34	2019-04-Mvpa	Average daily minutes of MVPA in April 2019.
35	2019-05-Mvpa	Average daily minutes of MVPA in May 2019.
36	2019-06-Mvpa	Average daily minutes of MVPA in June 2019.
37	2019-07-Mvpa	Average daily minutes of MVPA in July 2019.
38	2019-08-Mvpa	Average daily minutes of MVPA in August 2019.
39	2019-09-Mvpa	Average daily minutes of MVPA in September 2019.
40	2019-10-Mvpa	Average daily minutes of MVPA in October 2019.
41	2019-11-Mvpa	Average daily minutes of MVPA in November 2019.
42	2019-12-Mvpa	Average daily minutes of MVPA in December 2019.
43	2020-01-Mvpa	Average daily minutes of MVPA in January 2020.
44	2020-02-Mvpa	Average daily minutes of MVPA in February 2020.
45	2020-03-Mvpa	Average daily minutes of MVPA in March 2020.
46	2020-04-Mvpa	Average daily minutes of MVPA in April 2020.
47	2020-05-Mvpa	Average daily minutes of MVPA in May 2020.
48	2020-06-Mvpa	Average daily minutes of MVPA in June 2020.
49	2020-07-Mvpa	Average daily minutes of MVPA in July 2020.
50	2020-08-Mvpa	Average daily minutes of MVPA in August 2020.
51	2020-09-Mvpa	Average daily minutes of MVPA in September 2020.
52	2020-10-Mvpa	Average daily minutes of MVPA in October 2020.
53	2020-11-Mvpa	Average daily minutes of MVPA in November 2020.
54	2020-12-Mvpa	Average daily minutes of MVPA in December 2020.
55	2020-03-01-Mvpa	Average daily minutes of MVPA between March 1^st^-12^th^, 2020.
56	2020-03-13-Mvpa	Average daily minutes of MVPA between March 13^th^-31^st^, 2020.
57	2019-Mvpa	Average daily minutes of MVPA in 2019.
58	2020-Mvpa	Average daily minutes of MVPA in 2020.
59	2019-01-TEE	Average daily TEE (kcal) in January 2019.
60	2019-02-TEE	Average daily TEE (kcal) in February 2019.
61	2019-03-TEE	Average daily TEE (kcal) in March 2019.
62	2019-04-TEE	Average daily TEE (kcal) in April 2019.
63	2019-05-TEE	Average daily TEE (kcal) in May 2019.
64	2019-06-TEE	Average daily TEE (kcal) in June 2019.
65	2019-07-TEE	Average daily TEE (kcal) in July 2019.
66	2019-08-TEE	Average daily TEE (kcal) in August 2019.
67	2019-09-TEE	Average daily TEE (kcal) in September 2019.
68	2019-10-TEE	Average daily TEE (kcal) in October 2019.
69	2019-11-TEE	Average daily TEE (kcal) in November 2019.
70	2019-12-TEE	Average daily TEE (kcal) in December 2019.
71	2020-01-TEE	Average daily TEE (kcal) in January 2020.
72	2020-02-TEE	Average daily TEE (kcal) in February 2020.
73	2020-03-TEE	Average daily TEE (kcal) in March 2020.
74	2020-04-TEE	Average daily TEE (kcal) in April 2020.
75	2020-05-TEE	Average daily TEE (kcal) in May 2020.
76	2020-06-TEE	Average daily TEE (kcal) in June 2020.
77	2020-07-TEE	Average daily TEE (kcal) in July 2020.
78	2020-08-TEE	Average daily TEE (kcal) in August 2020.
79	2020-09-TEE	Average daily TEE (kcal) in September 2020.
80	2020-10-TEE	Average daily TEE (kcal) in October 2020.
81	2020-11-TEE	Average daily TEE (kcal) in November 2020.
82	2020-12-TEE	Average daily TEE (kcal) in December 2020.
83	2020-03-01-TEE	Average daily TEE (kcal) between March 1^st^-12^th^, 2020.
84	2020-03-13-TEE	Average daily TEE (kcal) between March 13^th^-31^st^, 2020.
85	2019-TEE	Average daily TEE (kcal) in 2019.
86	2020-TEE	Average daily TEE (kcal) in 2020.
87	2019-01-AEE	Average daily AEE (kcal) in January 2019.
88	2019-02-AEE	Average daily AEE (kcal) in February 2019.
89	2019-03-AEE	Average daily AEE (kcal) in March 2019.
90	2019-04-AEE	Average daily AEE (kcal) in April 2019.
91	2019-05-AEE	Average daily AEE (kcal) in May 2019.
92	2019-06-AEE	Average daily AEE (kcal) in June 2019.
93	2019-07-AEE	Average daily AEE (kcal) in July 2019.
94	2019-08-AEE	Average daily AEE (kcal) in August 2019.
95	2019-09-AEE	Average daily AEE (kcal) in September 2019.
96	2019-10-AEE	Average daily AEE (kcal) in October 2019.
97	2019-11-AEE	Average daily AEE (kcal) in November 2019.
98	2019-12-AEE	Average daily AEE (kcal) in December 2019.
99	2020-01-AEE	Average daily AEE (kcal) in January 2020.
100	2020-02-AEE	Average daily AEE (kcal) in February 2020.
101	2020-03-AEE	Average daily AEE (kcal) in March 2020.
102	2020-04-AEE	Average daily AEE (kcal) in April 2020.
103	2020-05-AEE	Average daily AEE (kcal) in May 2020.
104	2020-06-AEE	Average daily AEE (kcal) in June 2020.
105	2020-07-AEE	Average daily AEE (kcal) in July 2020.
106	2020-08-AEE	Average daily AEE (kcal) in August 2020.
107	2020-09-AEE	Average daily AEE (kcal) in September 2020.
108	2020-10-AEE	Average daily AEE (kcal) in October 2020.
109	2020-11-AEE	Average daily AEE (kcal) in November 2020.
110	2020-12-AEE	Average daily AEE (kcal) in December 2020.
111	2020-03-01-AEE	Average daily AEE (kcal) between March 1st–12t^h^, 2020.
112	2020-03-13-AEE	Average daily AEE (kcal) between March 13th–31st, 2020.
113	2019-AEE	Average daily AEE (kcal) in 2019.
114	2020-AEE	Average daily AEE (kcal) in 2020.
115	2019-01-LPA	Average daily minutes of LPA in January 2019.
116	2019-02-LPA	Average daily minutes of LPA in February 2019.
117	2019-03-LPA	Average daily minutes of LPA in March 2019.
118	2019-04-LPA	Average daily minutes of LPA in April 2019.
119	2019-05-LPA	Average daily minutes of LPA in May 2019.
120	2019-06-LPA	Average daily minutes of LPA in June 2019.
121	2019-07-LPA	Average daily minutes of LPA in July 2019.
122	2019-08-LPA	Average daily minutes of LPA in August 2019.
123	2019-09-LPA	Average daily minutes of LPA in September 2019.
124	2019-10-LPA	Average daily minutes of LPA in October 2019.
125	2019-11-LPA	Average daily minutes of LPA in November 2019.
126	2019-12-LPA	Average daily minutes of LPA in December 2019.
127	2020-01-LPA	Average daily minutes of LPA in January 2020.
128	2020-02-LPA	Average daily minutes of LPA in February 2020.
129	2020-03-LPA	Average daily minutes of LPA in March 2020.
130	2020-04-LPA	Average daily minutes of LPA in April 2020.
131	2020-05-LPA	Average daily minutes of LPA in May 2020.
132	2020-06-LPA	Average daily minutes of LPA in June 2020.
133	2020-07-LPA	Average daily minutes of LPA in July 2020.
134	2020-08-LPA	Average daily minutes of LPA in August 2020.
135	2020-09-LPA	Average daily minutes of LPA in September 2020.
136	2020-10-LPA	Average daily minutes of LPA in October 2020.
137	2020-11-LPA	Average daily minutes of LPA in November 2020.
138	2020-12-LPA	Average daily minutes of LPA in December 2020.
139	2020-03-01-LPA	Average daily minutes of LPA between March 1^st^-12^th^, 2020.
140	2020-03-13-LPA	Average daily minutes of LPA between March 13^th^-31^st^, 2020.
141	2019-LPA	Average daily minutes of LPA in 2019.
142	2020-LPA	Average daily minutes of LPA in 2020.
143	2019-01-MPA	Average daily minutes of MPA in January 2019.
144	2019-02-MPA	Average daily minutes of MPA in February 2019.
145	2019-03-MPA	Average daily minutes of MPA in March 2019.
146	2019-04-MPA	Average daily minutes of MPA in April 2019.
147	2019-05-MPA	Average daily minutes of MPA in May 2019.
148	2019-06-MPA	Average daily minutes of MPA in June 2019.
149	2019-07-MPA	Average daily minutes of MPA in July 2019.
150	2019-08-MPA	Average daily minutes of MPA in August 2019.
151	2019-09-MPA	Average daily minutes of MPA in September 2019.
152	2019-10-MPA	Average daily minutes of MPA in October 2019.
153	2019-11-MPA	Average daily minutes of MPA in November 2019.
154	2019-12-MPA	Average daily minutes of MPA in December 2019.
155	2020-01-MPA	Average daily minutes of MPA in January 2020.
156	2020-02-MPA	Average daily minutes of MPA in February 2020.
157	2020-03-MPA	Average daily minutes of MPA in March 2020.
158	2020-04-MPA	Average daily minutes of MPA in April 2020.
159	2020-05-MPA	Average daily minutes of MPA in May 2020.
160	2020-06-MPA	Average daily minutes of MPA in June 2020.
161	2020-07-MPA	Average daily minutes of MPA in July 2020.
162	2020-08-MPA	Average daily minutes of MPA in August 2020.
163	2020-09-MPA	Average daily minutes of MPA in September 2020.
164	2020-10-MPA	Average daily minutes of MPA in October 2020.
165	2020-11-MPA	Average daily minutes of MPA in November 2020.
166	2020-12-MPA	Average daily minutes of MPA in December 2020.
167	2020-03-01-MPA	Average daily minutes of MPA between March 1st–12th, 2020.
168	2020-03-13-MPA	Average daily minutes of MPA between March 13th–31st, 2020.
169	2019-MPA	Average daily minutes of MPA in 2019.
170	2020-MPA	Average daily minutes of MPA in 2020.
171	2019-01-VPA	Average daily minutes of VPA in January 2019.
172	2019-02-VPA	Average daily minutes of VPA in February 2019.
173	2019-03-VPA	Average daily minutes of VPA in March 2019.
174	2019-04-VPA	Average daily minutes of VPA in April 2019.
175	2019-05-VPA	Average daily minutes of VPA in May 2019.
176	2019-06-VPA	Average daily minutes of VPA in June 2019.
177	2019-07-VPA	Average daily minutes of VPA in July 2019.
178	2019-08-VPA	Average daily minutes of VPA in August 2019.
179	2019-09-VPA	Average daily minutes of VPA in September 2019.
180	2019-10-VPA	Average daily minutes of VPA in October 2019.
181	2019-11-VPA	Average daily minutes of VPA in November 2019.
182	2019-12-VPA	Average daily minutes of VPA in December 2019.
183	2020-01-VPA	Average daily minutes of VPA in January 2020.
184	2020-02-VPA	Average daily minutes of VPA in February 2020.
185	2020-03-VPA	Average daily minutes of VPA in March 2020.
186	2020-04-VPA	Average daily minutes of VPA in April 2020.
187	2020-05-VPA	Average daily minutes of VPA in May 2020.
188	2020-06-VPA	Average daily minutes of VPA in June 2020.
189	2020-07-VPA	Average daily minutes of VPA in July 2020.
190	2020-08-VPA	Average daily minutes of VPA in August 2020.
191	2020-09-VPA	Average daily minutes of VPA in September 2020.
192	2020-10-VPA	Average daily minutes of VPA in October 2020.
193	2020-11-VPA	Average daily minutes of VPA in November 2020.
194	2020-12-VPA	Average daily minutes of VPA in December 2020.
195	2020-03-01-VPA	Average daily minutes of VPA between March 1st–12th, 2020.
196	2020-03-13-VPA	Average daily minutes of VPA between March 13th–31st, 2020.
197	2019-VPA	Average daily minutes of VPA in 2019.
198	2020-VPA	Average daily minutes of VPA in 2020.
199	2019-01-SED	Average daily minutes of sedentary time in January 2019.
200	2019-02-SED	Average daily minutes of sedentary time in February 2019.
201	2019-03-SED	Average daily minutes of sedentary time in March 2019.
202	2019-04-SED	Average daily minutes of sedentary time in April 2019.
203	2019-05-SED	Average daily minutes of sedentary time in May 2019.
204	2019-06-SED	Average daily minutes of sedentary time in June 2019.
205	2019-07-SED	Average daily minutes of sedentary time in July 2019.
206	2019-08-SED	Average daily minutes of sedentary time in August 2019.
207	2019-09-SED	Average daily minutes of sedentary time in September 2019.
208	2019-10-SED	Average daily minutes of sedentary time in October 2019.
209	2019-11-SED	Average daily minutes of sedentary time in November 2019.
210	2019-12-SED	Average daily minutes of sedentary time in December 2019.
211	2020-01-SED	Average daily minutes of sedentary time in January 2020.
212	2020-02-SED	Average daily minutes of sedentary time in February 2020.
213	2020-03-SED	Average daily minutes of sedentary time in March 2020.
214	2020-04-SED	Average daily minutes of sedentary time in April 2020.
215	2020-05-SED	Average daily minutes of sedentary time in May 2020.
216	2020-06-SED	Average daily minutes of sedentary time in June 2020.
217	2020-07-SED	Average daily minutes of sedentary time in July 2020.
218	2020-08-SED	Average daily minutes of sedentary time in August 2020.
219	2020-09-SED	Average daily minutes of sedentary time in September 2020.
220	2020-10-SED	Average daily minutes of sedentary time in October 2020.
221	2020-11-SED	Average daily minutes of sedentary time in November 2020.
222	2020-12-SED	Average daily minutes of sedentary time in December 2020.
223	2020-03-01-SED	Average daily minutes of sedentary time between March 1st–12th, 2020.
224	2020-03-13-SED	Average daily minutes of sedentary time between March 13th–31st, 2020.
225	2019-SED	Average daily minutes of sedentary time in 2019.
226	2020-SED	Average daily minutes of sedentary time in 2020.

The “data raw.csv” dataset is the raw data used to generate the variables described in “data.csv”. In addition to daily values for MVPA, TEE, AEE, LPA, MPA, VPA, and sedentary time, minutes of non-wear time is available Table 3. gives a description for variables included in the “data raw.csv” dataset.

**Table 3:** Variable description, data raw.csv.

#	Variable	Description
1	ID	Anonymized participant identified (1–113).
2	Date	Date of data point. Date format: ‘mm.dd.yyyy’.
3	Steps	Number of steps on the specified date.
4	TEE	Number of total energy expenditure (in kcal) on the given date.
5	AEE	Number of activity energy expenditure (in kcal) on the given date.
6	Sedentary	Minutes of sedentary time on the given data. Not available for Garmin.
7	LPA	Minutes of light physical activity on the given date. Not available for Garmin.
8	MPA	Minutes of moderate physical activity on the given date.
9	VPA	Minutes of vigorous physical activity on the given date.
10	NonWear	Minutes of non-wear time on the given date. Not available for Garmin.
11	Provider	Provider name (Garmin/Fitbit).

## 2 Experimental Design, Materials and Methods

Participants who owned an activity tracker (or smart watch) from Fitbit, Garmin, or Oura (activity ring), and who agreed to share physical activity related data collected from these devices between January 2019 and December 2020, were eligible for inclusion. No participants owned an Oura.

Participants were recruited by publishing on UiT The Arctic University of Norway's web pages [3]. The story was picked up and published in national online news outlets [4,5] and in some closed internet forums. Recruitment was performed between October 2020 and December 2020.

Participants were asked to share data by authorizing data sharing from their activity tracker provider. Authorization and automatic data download were performed using mSpider, a tool which allow study participants to share daily activity tracker data automatically and continuously. The mSpider system is described elsewhere [1]. The mSpider tool also support data extraction from other providers, including Polar, Samsung, and Apple, but these providers could not be included in the study. Polar only allow prospective data extraction, and we could not extract activity data collected before participant signup date. Apple and Samsung require a custom application to be installed on the participant smartphone. These were not finalized before recruitment and could not be used.

The Norwegian COVID-19 lockdown was initiated March 12th, 2020, closing gyms, schools, universities, kindergartens, and similar institutions. People were asked to avoid groups as much as possible, but outdoor exercise was allowed. Social restrictions were gradually lifted in April and May, but gradually reintroduced from August 2020.

For each participant, historic data were downloaded from January 2019 up to the inclusion date. Prospective data were download daily, up to and including December 2020. Authorization information was removed in January 2021, and data access to provider systems were no longer available.

For several participants, PA data were partly unavailable for the full two-year period. This was due to two reasons; (1) the participant did not own the device in the beginning of the period, and (2) the participant did not wear the device for a period of time. For the former, no PA values were registered. The latter were indicated with a zero value for all PA outcomes. The “data raw.csv” file (i.e., daily values) includes all days of 2019 and 2020 for each participant where data were available from the provider, also for the days where the participant did not wear the device.

In the “data.csv” file (i.e., averages), valid days were used to calculate monthly and yearly averages for each participant. The value ‘NULL’ indicates that no valid days existed in the specified period for the given data type. Valid days were defined as days with at least 10 h of wear time [6], or more than 150 steps. A step threshold was set because wear time was not available for Garmin devices. Since Fitbit provides both daily steps and hours of wear time, the 150-threshold were selected by identifying the lowest step count among Fitbit-participants where wear time was more than 10 h.

## Ethics Statements

Participants had to actively enrol in the data collection by authorizing access to their activity tracker provider's online data storage. Participants were informed that authorizing access constituted informed consent. The authors have the right to redistribute the data.

The study was reviewed by The Regional Committees for Medical and Health Research Ethics North (reference 164780). The data collection was reviewed by Norwegian Centre for Research Data (reference 628485).

## Data Availability

Replication Data for: Dataset of Consumer-Based Activity Trackers as a Tool for Physical Activity Monitoring in Epidemiological Studies During the COVID-19 Pandemic (Original data) (Dataverse).

## CRediT authorship contribution statement

**André Henriksen:** Conceptualization, Software, Investigation, Data curation, Formal analysis, Writing – original draft. **Erlend Johannessen:** Software, Investigation, Writing – review & editing. **Gunnar Hartvigsen:** Conceptualization, Writing – review & editing, Supervision. **Sameline Grimsgaard:** Conceptualization, Writing – review & editing, Supervision. **Laila Arnesdatter Hopstock:** Conceptualization, Writing – review & editing, Supervision.

## Declaration of Competing Interest

The authors declare that they have no known competing financial interests or personal relationships that could have appeared to influence the work reported in this paper.
